# Personal sustained cooperation based on networked evolutionary game theory

**DOI:** 10.1038/s41598-023-36318-7

**Published:** 2023-06-05

**Authors:** Jun Yan

**Affiliations:** School of Public Finance and Economics, Shanxi University of Financial and Economics, Taiyuan, 030006 China

**Keywords:** Network topology, Evolutionary theory

## Abstract

Evolutionary game theory on complex networks provides an effective theoretical tool to explain the emergence of sustained cooperative behavior. Human society has formed various organizational networks. The network structure and individual behavior take on a variety of forms. This diversity provides the basis for choice, so it is crucial for the emergence of cooperation. This article provides a dynamic algorithm for individual network evolution, and calculates the importance of different nodes in the network evolution process. In the dynamic evolution simulation, the probability of the cooperation strategy and betrayal strategy is described. In the individual interaction network, cooperative behavior will promote the continuous evolution of individual relationships and form a better aggregative interpersonal network. The interpersonal network of betrayal has been in a relatively loose state, and its continuity must rely on the participation of new nodes, but there will be certain "weak links" in the existing nodes of the network.

## Introduction

The management of interpersonal relationships has been a long-standing concern among scholars. Porter and Woo^1^ and Wolff and Moser^2^ suggest that strategic management of personal social relations can effectively enhance an individual's ability to acquire social resources. This involves establishing and maintaining informal connections, commonly referred to as the construction of personal social networks^3^. For example, in the personal professional field, contact with leaders and colleagues helps employees improve work efficiency and performance^4^.

The traditional approach to studying social networks assumes that they are static and predetermined, known as structural determinism^5^. However, recent research has challenged this view, suggesting that individuals can actively shape, maintain, and expand their social network connections through their own active behaviors^6–9^.

The heterogeneity of individual relationships in real life makes the individual interaction network have a rather complex topology, similar to a complex network described by Nowak^10^. In this network, individuals are represented as nodes, and the interactions between them are represented as edges connecting these nodes.

According to Gibson^11^, the network topology can be goal-oriented, where individuals act by creating and nurturing relationships among specific individuals. Kuwabara^12^ further emphasized the importance of actively maintaining informal working relationships to achieve individual career development goals. This ability is crucial for individuals to gain important information and cooperation from external sources^13^. Establishing social relations with specific objects involves developing individual connections for social subjects with specific social resources^3^. Actively screening contacts can effectively save social communication costs^14^. It is important to focus on the content of the contact, which should involve exchanging information, interests, and influence^15^, as well as exchanging social resources held by each other^16^.

The individual network construction process involves dynamic cooperative network organization based on membership interaction with adjacent individuals on the spatial grid, which evolves with social development. People obtain different benefits by choosing different learning strategies. In the context of social networks, the way people communicate and share information is influenced by their relationships with others in their network. This relationship is subject to evolution rules and behavior strategies. The clustering coefficient (*cc*) is a measure of the strength of the connection between two adjacent nodes in the network. The level of interconnectivity between neighboring points of a node in a human interaction network gives a network with certain characters. Such networks are patterns of connections among individuals within a group, and are characterized by dynamic mechanisms^16^.

This paper examines how individuals establish their social communication network in a dynamic social environment. We utilize the cluster coefficient to describe the growth process and have discovered some fascinating phenomena. Firstly, individuals tend to adopt cooperative strategies in the long-term process of building interpersonal networks. Secondly, stronger trust foundations between individuals lead to greater cohesiveness under cooperation strategies. Finally, to maintain interpersonal networks under the betrayal strategy, it is essential to continuously add new nodes to the network.

This paper is organized into five sections. Section "Related literature" provides a review of related literature. In section "Methodology", we describe the methodology and iterative algorithm used and analyze the cohesion of interpersonal network mechanisms under different strategy choices (cooperation and betrayal). Section "Discussion" presents the equilibrium analysis and supporting arguments. Finally, Section "Conclusion" concludes the paper.

## Related literature

### Cooperation behavior in human society

Cooperative behavior is prevalent in both nature and human society, making it a crucial area of study. Game theory serves as a valuable tool for examining how individuals interact with one another. By incorporating the concept of bounded rationality, game theory has become an even more effective means of explaining real-world interactions between individuals. Smith and Price^17^ introduced the concept of natural selection and variation from evolutionary theory into game theory, proposing the theory of evolutionary game. This theory suggests that individuals with bounded rationality learn and improve their adaptability through repeated games, ultimately aiming to optimize their profits and achieve a stable equilibrium^16^. Nowak^17^ proposed an evolutionary stability strategy for a finite population, while Grunert^18^ studied the existence of a stable evolution strategy in ecological dynamics.

To achieve a common goal, individuals must establish a cooperative relationship. However, challenges may arise during the process of individual interaction, which can affect the final outcome of cooperation^19^. In order to ensure the sustainability of cooperation, certain mechanisms must be put in place to encourage even the most selfish individuals to participate^20^.

### The role of the reciprocity mechanism in human society

Cooperative behavior is usually considered altruistic behavior because it requires individuals to pay a certain cost to provide benefits for the other party, and the betrayer will gain benefits by refusing to pay the cost, which can achieve the result of "getting something for nothing". Therefore, individual cooperative behavior often faces the prisoner's dilemma^21^.

Despite the fact that betrayal is a calculated decision made in one's own self-interest, there are still numerous instances of cooperative behavior observed in everyday life. Evolutionary biology has identified several mechanisms that may account for such behavior^22^, including kinship selection^23^, direct reciprocity^24^, indirect reciprocity^25^, and network reciprocity^26,27^.

Network reciprocity is an effective mechanism to explain the evolution of cooperation in social and biological systems. In these systems, populations are structured networks with various restrictions and constraints. By employing network reciprocity, individuals can optimize the system as a whole^28–32^. Given the specific network topology, complex networks can modify social dilemmas^33^.

### Repetitive cooperation and bonding performance

A well-designed interactive network can enhance the information processing and learning outcomes^31^. Through this network, one can identify learners’ characteristics like working style, cognitive ability, and hobbies^34^, and find the most compatible learning partner based on personal traits. This means that individuals with similar characteristics are more likely to engage in repeated and in-depth interpersonal communication, leading to better learning outcomes. Even in a professional organization, cultivating good interpersonal relationships can have a positive effect on communication efficiency. Game theory has been utilized to simulate social behavior^35–37^, specifically in understanding the mechanisms of heterogeneous and dynamic interpersonal interaction. Studies have shown that closer relationships within a network tend to have longer durations of interaction and higher frequencies^38^ compared to more distant relationships^39–41^. Moreover, the weighted network can be used to simulate participants' social preference behavior^42^. With a proper evolution rule^43^, the weighted network can also be used to simulate the function of interpersonal relationship dynamic change.

The utilization of social preference mechanism in network learning has a positive impact on promoting sustained cooperation as it aids in resisting the temptation of betrayal. The efficiency of group cooperation has obviously increased in continuous interactions. As communication within an organization becomes more frequent, there is a potential for the network to become less receptive to new information. This lack of new information can result in a lack of diversity in the proper cognition of problems^44^, leading to a decrease in the effectiveness of information learning due to reduced diversity.

The literature research highlights the significance of interpersonal interaction networks in facilitating human cooperative relationships. Game theory can be employed to investigate the formation of these networks and the cooperative behavior within them.

## Methodology

This paper presents a growth model for collaborative networks, which follows an iterative rule for each grid in the social network. The concept of a random graph was introduced by Erdos and Rényi in their 1959 seminal article, and we follow this tradition by considering a game interaction network in the following form:

1 $$G=(V, E,X)$$

G is a series of games and related relations in interactive cyberspace, The node set in this space represents each person in the cyberspace, denoted by V = (v_1_, v_2_…, v_n_), and edge set E denotes the relationships for each node in the interactives. The trust weighted matrix X = X_ij_ (n × n) represents relationships between individual i (node i in the concerning network) and j (node j in the concerning network), described as trust foundations between individuals in the network (which is described as trust foundations between individuals in the network), which evolves according to certain rules. In an undirected network, a node's degree is determined by the number of edges directly connected to it. The average degree of the network (*k*) is calculated as the average value of the degrees of all nodes in the network. In a directed network, a node's degree is determined by both its egress and ingress. Egress refers to the number of edges from this node to other nodes, while ingress refers to the number of edges from other nodes to this node. Our network evolution mechanism is straightforward. Each participant interacts with their neighboring grids in all directions, without double counting, to gain closer relationships and accumulated knowledge stock. If either party employs a defect strategy, it will lead to the termination of the cooperation relationship and the loss of the social intimacy already gained.

This model uses a weighted network to simulate the process of individual interaction’s evolution with the following rule:

$${X}_{ij}^{t}=\left\{\begin{array}{ll}{X}_{ij}^{t-1}(1+\lambda )&\quad {v}_{i}={v}_{j}=c \quad(2)\\ \lambda -{\left(1-\lambda \right)X}_{ij}^{t-1}&\quad {{v}_{i}\ne {v}_{j}}_{i} \,\,\,\quad\quad(3)\\ {X}_{ij}^{t-1}&\quad {v}_{i}={v}_{j}=d \quad(4)\end{array}\right.$$

In this network, ${X}_{ij}^{t}$ is used to represent an individual's social reputation based on their behavior history, while λ represents the time value of one person. If a node has not exhibited any behavior throughout their lifespan, their reputation will gradually accumulate over time. This is similar to the way elderly individuals are respected in society, even if they have not recently demonstrated any notable behavior. Naturally, we use t to represent different periods of network evolution. Therefore, the reputation of the next period has a time value compared with that of the current period, which is represented as Formula (2):

2 $${X}_{ij}^{t}=(1+\lambda ){X}_{ij}^{t-1}$$

The formula (2) represents a time difference between the trust foundation of the previous period (t-1) and the trust foundation of the current period (t). This means that due to the smooth progress of cooperation, the social evaluation of a node remains consistent with the original evaluation. Therefore, the time value will continue to accumulate until the node leaves the network, which typically occurs when a person passes away and their social network ends. If cooperation fails in a certain period, it may be due to both parties choosing to betray simultaneously or due to inconsistent strategic choices (i.e., a defector). If both parties choose not to cooperate simultaneously, then no one suffers losses, which is represented by formula (4).

In simpler terms, formula (4) shows that both parties have rational expectations regarding their cooperation, resulting in the relationship between them remaining unchanged and ${X}_{ij}^{t}$ remaining constant. However, if one of the parties defects, they will be punished by losing the trust foundation established through previous cooperation and their original social evaluation. This is represented by formula (3). Formula (3) in the prisoner's dilemma game involves one party choosing to betray while the other party chooses to cooperate. This strategic choice leads to a betrayal cost for the cooperating party, ultimately damaging the trust foundation between the two parties (node i and node j). In period t, the trust foundation ${X}_{ij}^{t}$ between the two parties in this article has been completely destroyed, resulting in the termination of their cooperative relationship. In order to address the issue of a cooperator's loss of time value, it is necessary to consider the deduction of the initial cognition. Additionally, formula (3) includes a positive component on the right-hand side that represents the previous evaluation of the node within the network. This implies that although the trust between nodes may have been compromised, a node's past positive reputation can partially excuse its behavior. This phenomenon can be used to explain why individuals are more conscious of their actions and reputation within their own clique.

Evolutionary game theory is a field that studies the evolution of a group over time and analyzes the coordination between technological progress^45^ and economic growth^46^. It applies the principles of biological fitness and natural selection to explain the equilibrium evolution of mutation mechanisms^47,48^. Furthermore, Evolutionary game theory can be used to understand the dynamic evolution process of individual learning as an ESS^49–53^, as many scholars have argued. In recent years, network evolutionary game theory has been widely applied to social network research questions. Lee et al.^54^ used this approach to examine the threat of corruption to social stability. Hilbe et al.^55^ discussed direct reciprocity mechanisms within this framework, while Schmid et al.^56^ explored the working mechanisms of both direct and indirect reciprocity and their relationship. In a study on the impact of individual heterogeneity on cooperation, Hauser et al.^57^ found that extreme inequality can lead to the unsustainability of cooperative behavior. However, if the sources of individual heterogeneity are consistent, it can enhance the welfare level of the entire cooperative network. Martinangeli et al.^58^ argued that social inequality has an impact on social cooperative behavior by influencing people's sense of identification with their own network, and individuals who has a strong identification with their social status are more likely to exhibit sustainable cooperation. Wang et al.^59^ presented a network evolutionary game theory framework that highlighted the importance of equal individual costs in maintaining cooperative behavior within social networks. The above literature all indicate the important tool significance of network evolutionary game theory in studying social reality issues. In various real-world complex network systems, including biological, social, technological, and other networks, scale-free and clustering characteristics are present. These characteristics are crucial in the network's dynamic process. By comprehending the interplay between game dynamics and the underlying structure, we can gain a better understanding of how cooperation emerges within a group. In the network settings provided, the strategy used by each node in the game is represented by G. Each node in the network represents an agent, and the edge represents the game interaction between agents. The networks constructed through numerical simulation under cooperation and betrayal strategies are based on the network relationship given in formula (1).

### Cooperation strategy

We utilize the evolutionary paradigm of evolutionary game theory to examine the mechanism of human interactive behavior. The interactions between agents are limited and typically occur in small, local areas. Within a group, fixed agents make contact within a specific time frame. The study of evolutionary games on networks is also referred to as network evolutionary games or network reciprocity. Network reciprocity is a crucial mechanism in the emergence of cooperative behavior in complex systems. The Monte Carlo simulation experiment follows a basic evolution process. 1st step, at each time step, the agent interacts with all its neighbors in the game. 2nd step, the related parties cumulatively obtain gains from all game interactions. 3rd step, an iteration rule given by formula (2) is applied to update the trust foundation ${X}_{ij}^{t}$ to the next period t. Last step, the iterative evolution is repeated until the simulation ends. Once two nodes establish a cooperative relationship, they create a private trust foundation between themselves. This means that individual nodes do not need to rely on the network's evaluation of their interactive partners in order to make behavior decisions. Therefore, Eq. (2) does not include network evaluation. Partner selection is the initial step towards cooperative behavior among nodes. The selection process can be based on an existing trust network where the trustworthiness of a node is evaluated by the network. However, this process can be complex as the evaluation of a node's trustworthiness in a family trust network may not only depend on its own behavior but also on the trustworthiness of its relatives for example. This paper explores the creation and evolution of individual networks composed of nodes that share similar characteristics. When assigning a trust basis to newly added nodes in our network, we utilized a unit value of 1. This approach enabled us to track the evolution of trust basis and network characteristics between nodes, as previously discussed and presented in our algorithm.

**Figure 1:**
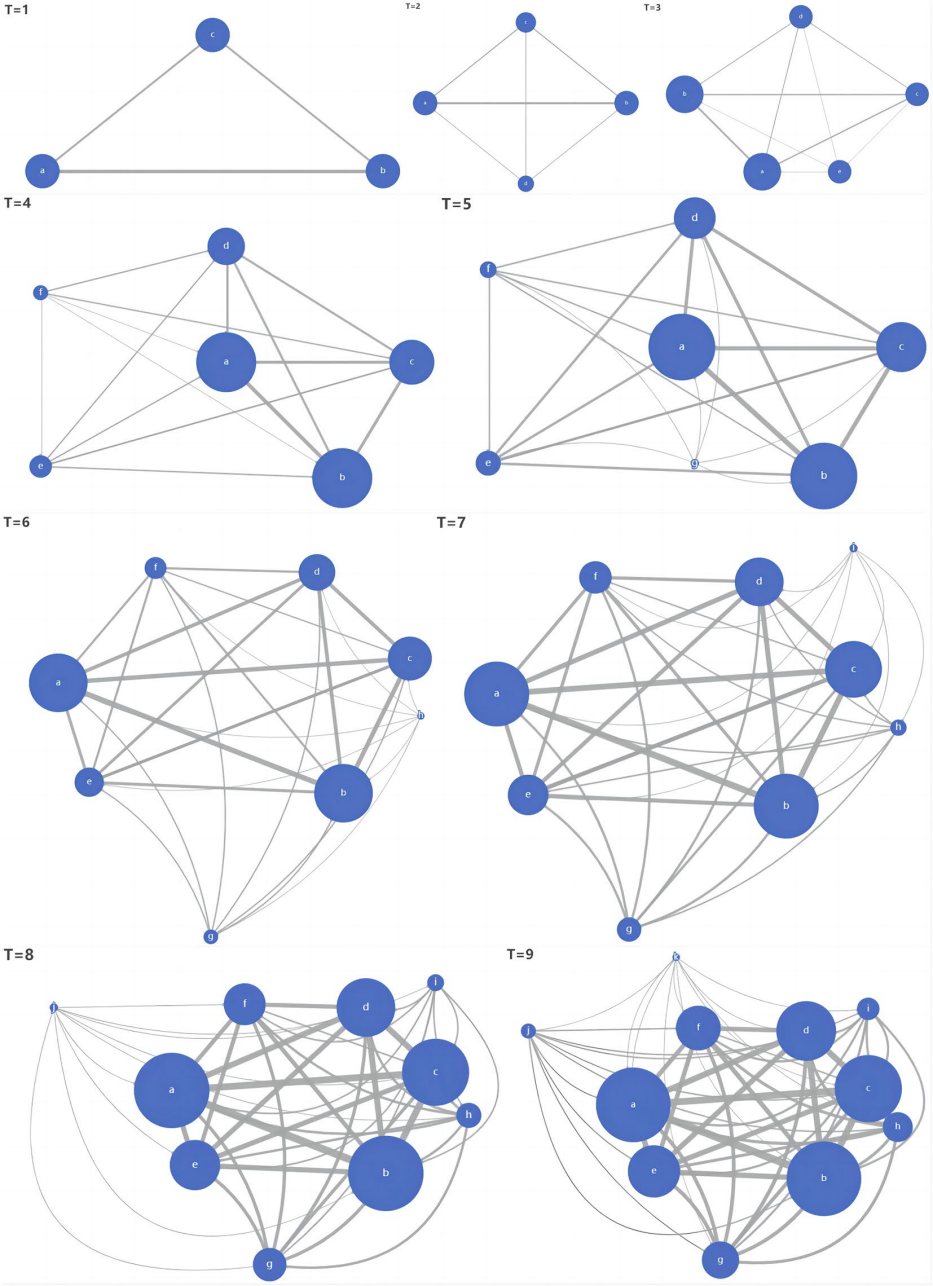
Evolution of the individual interaction network under the cooperation strategy. Node importance is distinguished by node size, in which larger represents more importance and the shade of the line represents the importance of the connection in the network evolution.

**Figure 2:**
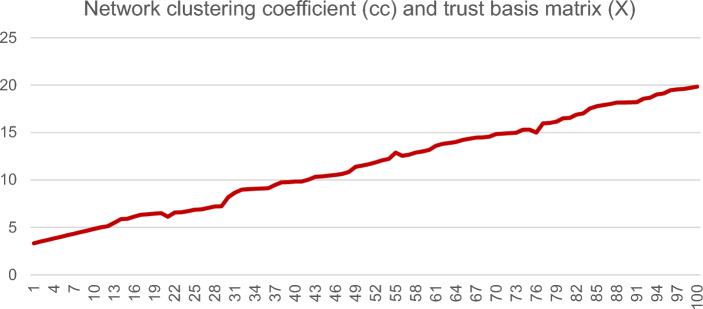
Evolution of the individual interaction network under the cooperation strategy. Network clustering coefficient and trust basis matrix.

Figure 1 illustrates the first nine steps of Monte Carlo simulation, assuming all nodes in the network adopt the 'cooperation' strategy. However, it is important to note that this assumption of adding a large number of nodes in each period is unrealistic. In reality, there may be a varying number of nodes added in the same time period or no nodes added at all. Additionally, there may be more than two nodes that generate cooperation. For the purpose of this study, we have simplified the network by only considering the scenario where two nodes conduct cooperation. In this simulation, we ran a total of 1000 periods. However, due to the dense nature of the resulting network, we only presented the results for the first 9 iterations. Our goal was to analyze the structure and evolution of human networks with a fixed trust foundation (${X}_{ij}^{t}$) and time value ($\lambda$). Each dot in the network represents a node, and the size of the circle represents the node degree based on the importance of all the nodes in the network (centrality of nodes).In this iterative network, we utilize closeness centrality to gauge the significance of each node, as demonstrated in Table 1. This metric determines the ease with which a node can communicate with other nodes in the network, thereby indicating its importance. As the interpersonal network evolves, the initially established nodes gradually gain significance with each iteration, ultimately becoming the most essential nodes in the network. The significance of newly added nodes in a network is determined by their initial relationships with the existing nodes. Stronger trust between nodes will result in those nodes becoming more important during subsequent iterations. As such, understanding the function and mechanism of trust foundation in interpersonal networks is of great interest. To investigate the trust foundation in interpersonal networks, we assigned varying trust foundation values to the initial nodes of the network. Figure 2 depicts the relationship between network cohesion and trust foundation. The results show that a greater trust basis leads to higher cohesion in the individual interaction network.

**Table 1:** The centrality of each node of the interpersonal network in 9 steps under the cooperation strategy.

Closeness centrality measures		
	1	2
	Farness	nCloseness
2 b	10.000	100.00
1 a	11.000	90.909
3 c	11.000	90.909
9 i	11.000	90.909
7 g	11.000	90.909
6 f	11.000	90.909
8 h	11.000	90.909
5 e	12.000	83.333
4 d	12.000	83.333
10 j	12.000	83.333
11 k	12.000	83.333

Statistics		
	1	2
	Farness	nCloseness
1 Mean	11.273	88.981
2 Std Dev	0.617	4.966
3 Sum	124.000	978.788
4 Variance	0.380	24.664
5 SSQ	1402.000	87364.555
6 MCSSQ	4.182	271.309
7 Euc Norm	37.443	298.575
8 Minimum	10.000	83.333
9 Maximum	12.000	100.000
Network centralization=25.59%		

In 1992, Nowak^60^ introduced spatial structure into evolutionary game theory, where the grid interacts with surrounding nodes. The research demonstrated that in contrast to the annihilation and betrayal of cooperative behavior in a uniformly mixed population, when actors are restricted by network structure, cooperative clusters emerge in the grid network. These cooperative groups can resist the temptation of betrayal. These cooperative clusters will evolve over time, but they are not inherently stable and can be easily disrupted by minor events.

Previous studies have shown the significance of cooperative strategies in maintaining network stability. The frequency of these strategies within a network acts as a crucial indicator for stable network relationships. In fact, some researchers have found that the frequency of cooperative strategies in a steady-state remains unaffected by the initial distribution of the payoff^61^.

### Betrayal strategy

**Figure 3:**
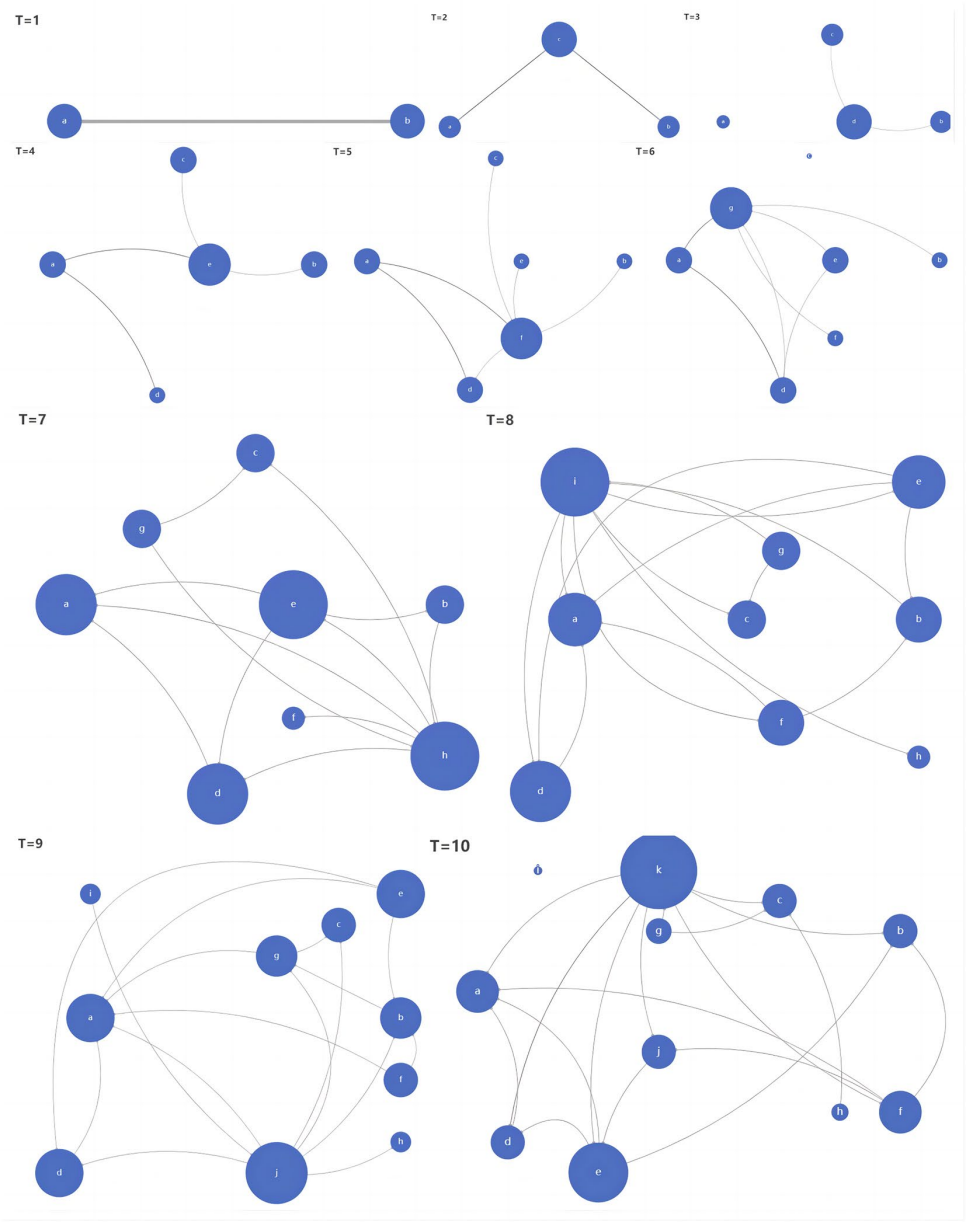
Evolution of the individual interaction network under the betrayal strategy. Node importance is distinguished by node size, in which larger represents more importanceand the shade of the line represents the importance of the connection in the network evolution.

Let us assume that nodes adopt the "betrayal" strategy when interacting. In this simulation, nodes are assumed to adopt the 'betrayal' strategy when interacting. The simulation follows the same basic idea as the 'cooperation' networks described above, where interactions occur between two nodes and a new node is added in the following phase. Additionally, the results of the iterated game between nodes from the previous phase are displayed in this phase using formula (3). For this study, we utilized a unit value of 1 in the cooperation network. Through our analysis of the evolutionary process, we observed that as nodes joined in phase t, their connections would disappear in phase t + 1, ultimately becoming remote nodes in the network. By introducing new nodes, we were able to track the development of the interaction network. Figure 3 displays the network organization status after 9 iterations.

Our research indicates that every new node added to the network holds significant importance. However, we have observed that existing nodes often become isolated and the survival of the entire network depends on the joining of new nodes. Interestingly, we have also discovered that if there is no relationship between the new access node and the existing nodes during the current period, it may result in a 'weak link' in the evolution process of the second phase. For example, in the third phase, there was no relationship between node a and node d. However, a connection was formed in the fourth phase and still existed at t = 10. This connection made node a an important node in the loose network system. Furthermore, this connection became increasingly connected in subsequent iterations.

This may because that the new node learned the behavior style of the pre-existing node during their initial interaction of existing in a same network. And for some reasons, these nodes have developed an actual interaction, then both nodes have rational expectation of this connection, that is, the formula (4) applies. This establishes a stable relationship between the two nodes, making them both crucial connection nodes in this delicate network. In the continuous betrayal strategy, the trust basis does not affect the cohesion of the network in each iteration. As a result, the network cluster coefficient remains at a consistently low level, with a value of less than 1 in our numerical simulation. This indicates that the overall network is highly fragile.

Szolnoki^62^ discovered that exploitation strategies can evolve in a stable manner within grid networks. These strategies can also serve as a 'Trojan horse' to promote cooperation within the network. However, the use of betrayal strategies in interpersonal interaction networks can lead to fragile relationships within the network.

Our simulation process revealed that the network remains fragile regardless of the trust foundation between nodes. The results of simulations on cooperative and betrayal strategies indicate that betrayal is more frequent than cooperation in interpersonal networks over the long run. Hanaki et al.^63^ suggested that individuals in social networks use cost-effectiveness algorithms to choose their cooperative relationships and partners, and indicated that high-quality cooperative relationships can develop even in loosely connected networks during the iteration process. Han et al.^64^ discovered that the structure of a network plays a crucial role in the development of cooperative strategies in complex network games over a long period of time.

## Discussion

Social relations play an important role in facilitating cooperation among individuals. The trust weighted matrix ${X}_{ij}^{t}$ serves as the foundation for an indirect reciprocity mechanism in interpersonal networks, specifically through reputation. While traditional reciprocity is based on the principle of 'give and you shall receive,' indirect and network reciprocity mechanisms are more prevalent in human social networks. Our organization donates to charity without expecting anything in return. However, we believe that individuals who contribute positively to society hold a certain level of reputation and are considered valuable members in our society^17^. Studies have shown that indirect reciprocity plays a crucial role in promoting cooperative behavior within human society^65–72^. This article utilizes network evaluation of individual nodes to determine their 'social value'. Studies have shown that indirect reciprocity can effectively address the free-ride problem^73^. Trust formation is influenced by the weighted matrix values, indicating our willingness to collaborate with others. Blood relationships are a significant source of trust, and early experiences of cooperation can also contribute to its development.

In the mechanisms of indirect reciprocity and network reciprocity, reputation and status^74^ have been established in our society. This can be compared to a 'social seal'^39^, where unfamiliar individuals are labeled to assess the potential benefits and risks of cooperating with them. Our study found that social reputation operates in various forms, including the deep trust foundation between a father and son due to their direct blood bond. In this kind of relationship, regardless of one party's strategy, the other party is willing cooperate, even it means the cost of trust. This foundation of trust makes achieving cooperation in these types of relationships much easier.

In order for cooperation to occur under the mechanisms of indirect reciprocity and network reciprocity, participants must evaluate their imaginary players^74^. Since the players on both sides do not directly interact and are separated by a large gap in space and time, the benefits of the game cannot be realized during interaction. According to Nowak^75^, cooperation through this mechanism involves both parties and is also influenced by the social reputation of the 'onlooker' or witness of the payer in society. The presence of this social reputation puts the payer in a favorable position for potential cooperation opportunities, as they are seen as individuals who pay more and gain more social capital, ultimately adding more value to their existence in society.

Alexander^76^ suggests that indirect reciprocity and the network reciprocity mechanism may form the foundation of our moral system. By utilizing these mechanisms, network organizations with strong social connections can avoid 'free-rider' behavior^75,76^. In a closed social environment, the historical information of crowd cooperation can be used to assess a person's 'reliability' and value. This highlights the significance of initial nodes in the network when implementing a cooperation strategy (Fig. 1).

Establishing a positive social reputation is crucial for improving one's fitness within a complex social network. In such networks, it may be impossible for others to have a complete understanding of an individual's cooperation history. As a result, social reputation becomes an integral aspect of an individual's social capital. When this understanding is a social norm, individuals are motivated to actively maintain their reputation within the system. In addition, individuals in a social network assign a score to each node in their network to assess their willingness to cooperate. This process creates a self-reinforcing positive feedback loop, where individuals with higher reputations receive more cooperation opportunities and accumulate more social capital. This phenomenon, known as social capital, is more prevalent in networks with close connections, indicating a deeper foundation of trust ${X}_{ij}^{t}$.This type of connection between groups can be viewed as an informal contract that is not necessarily documented, and social capital is often transferred through word of mouth. Those individuals who have a positive reputation are likely to receive higher evaluations from others, while those with a negative reputation may be viewed as undesirable partners, resulting in social punishment. Although this punishment may not be legally enforced, within this relatively closed social system, it can have serious consequences. In general, when there is a small foundation of social trust, betrayal strategies tend to be more prevalent. This is because, due to a lack of information, individuals can avoid punishment from informal social organizations even if they do not adopt cooperative strategies. As a result, the more structured a network is and the more individuals value their social reputation within that network, the more cohesive the network becomes. This argument is reflected in the simulation above.

The existence of a blood relationship can provide a good reason for trust. Even if the other person has a questionable social reputation, when working with a family member, the decision to betray them will have a negative impact on their own genetic survival. This concept has been studied by Binmore^77^ and Hamilton^61^, and Haldane has proposed that 'I will jump into the river to save two brothers or eight cousins' is a cooperative screening algorithm based on genetic relationships.

The reputation of a social network has a significant impact on how each individual node is evaluated externally. As a result, each node in the network takes an active role in maintaining its own reputation, which contributes to a cohesive group behavior. The connections within the network are formed through a variety of social interactions between nodes, creating a dynamic and complex system.

Granovetter's work^78^ elaborates on the theory of strong and weak social connections, which suggests that the strength of connections within a small group is higher than that between the group and the outside world. Aiello^79^ used a model of ten social relation dimensions from social psychology to evaluate social tie strength and found that high-quality social connections can effectively enhance an individual's economic opportunities and performance.Chetty^80^ argued that there exists a notable correlation between the rise of social capital and the robustness of social interaction networks. Melamed^81^ showed that the cooperative structure of social networks and the asymmetry of social capital reinforce each other, ultimately resulting in systemic inequality within society. Within this article, the terms 'strong connection' and 'weak connection' are used to describe the varying levels of intensity present within interpersonal interaction networks. Interpersonal relationships are closely linked to an individual's endowments and personal preferences. Weak connections indicate a fragile cooperative relationship between individuals, such as those who ride a bus together, watch a movie in the same cinema, or dine together in the same restaurant. Although they may not have actual interpersonal interactions, they still follow the same social norms at the same time, thus adhering to a social norm as a whole. We hypothesize that conforming to social norms can improve an individual's fitness within social networks, and this requires further investigation. The act of actively and rationally expanding one's social connections can lead to stronger connections. Strong connections are the result of collaborative behavior between individuals, and successfully establishing them can increase the social capital stock of nodes.

The size of a social network is dependent on the number of participating nodes and the intensity of interaction. Consequently, it would be worthwhile to investigate the characteristics of economic organizations, such as cities and enterprises, as well as social networks, that attract external nodes and foster strong connections. This research direction presents an interesting avenue for exploration.

## Conclusion

In a human social network, individuals belong to different social circles and communicate with various people. Successful cooperation relies not only on the significance of the task at hand but also on the willingness and understanding of both partners. This cognitive process takes time and requires repeated communication. However, once a person's social reputation is established, the likelihood of successful cooperation is determined.

In interpersonal communication and cooperation, an individual's weight matrix serves as their 'signboard'. It is this 'social label' that determines whether they are worthy of our cooperation and whether they are an appropriate candidate to complete a task. Thorough research and investigation on the cooperative object are not always feasible in a cooperative setting. Various interaction algorithms may result in different modes of cooperation; however, it is imperative to elucidate the cooperative behavior of human networks in any research. This is essential to comprehend the cooperative essence of informal human organizations.

## Data Availability

The datasets used and/or analysed during the current study available from the corresponding author on reasonable request.

## References

[1] Porter CM, Woo SE (2015). Untangling the networking phenomenon: A dynamic psychological perspective on how and why people network. J. Manag..

[2] Wolff HG, Moser K (2006). Entwicklung und validierungeinernetworkingskala [Development and validation of a networking scale]. Diagnostica.

[3] Forret ML, Dougherty TW (2001). Correlates of networking behavior for managerial and professional employees. Group Org. Manag..

[4] Brennecke J (2019). Dissonant ties in intraorganizational networks: Why individuals seek problem-solving assistance from difficult colleagues. Acad. Manag. J..

[5] Emirbayer M (1998). Agent-philosophy; sociology–philosophy; action-theory. Am. J. Sociol..

[6] Tasselli S, Kilduff M (2021). Network agency. Acad. Manag. Ann..

[7] Bensaou BM, Galunic C, Jonczyk-Sédès C (2014). Players and purists: Networking strategies and agency of service professionals. Organ. Sci..

[8] Kilduff M, Krackhardt D (1994). Bringing the individual back in: A structural analysis of the internal market for reputation in organizations. Acad. Manag. J..

[9] Kilduff M, Brass DJ (2010). Organizational social network research: Core ideas and key debates. Acad. Manag. Ann..

[10] Nowak MA, Sigmund K (1992). Tit for tat in heterogenous populations. Nature.

[11] Gibson C, Hardy JH, Ronald Buckley M (2014). Understanding the role of networking in organizations. Career Dev. Int..

[12] Kuwabara K, Hildebrand CA, Zou X (2018). Lay theories of networking: How laypeople’s beliefs about networks affect their attitudes toward and engagement in instrumental networking. Acad. Manag. Rev..

[13] Wolff HG, Moser K, Grau A, Deller J (2008). Networking: Theoretical foundations and construct validity. Readings in Applied Organizational Behavior from the Lüneburg Symposium—Personality at Work.

[14] Wolff HG, Kim S (2020). The costs of networking in nonwork domains: A resource-based perspective. Career Dev. Int..

[15] de Janasz SC, Dowd KO, Schneider BZ (2018). Interpersonal Skills in Organizations.

[16] Cropanzano R, Mitchell MS (2005). Social exchange theory: An interdisciplinary review. J. Manag..

[17] Smith JM, Price GR (1973). The logic of animal conflict. Nature.

[18] Nowak MA, Sasaki A, Taylor C (2004). Emergence of cooperation and evolutionary stability in finite populations. Nature.

[19] Grunert K, Holden H, Jakobsen ER (2021). Evolutionarily stable strategies in stable and periodically fluctuating populations: The Rosenzweig–MacArthur predator–prey model[J]. Proc. Natl. Acad. Sci..

[20] Panait L, Luke S (2005). Cooperative multi-agent learning: The state of the art. Auton. Agent. Multi-Agent Syst..

[21] Ephrati E, Rosenschein JS (1996). Deriving consensus in multiagent systems. Artif. Intell..

[22] Macy MW, Flache A (2002). Learning dynamics in social dilemmas. Proc. Natl. Acad. Sci. USA.

[23] Nowak MA (2006). Five rules for the evolution of cooperation. Science.

[24] Hamilton W (1964). The genetical evolution of social behavior. J. Theor. Biol..

[25] Trivers LR (1971). The evolution of reciprocal altruism. Q. Rev. Biol..

[26] Nowak MA, Sigmund K (2005). Evolution of indirect reciprocity. Nature.

[27] Nowak MA, May RM (1992). Evolutionary games and spatial chaos. Nature.

[28] Ohtsuki H, Hauert C, Lieberman E (2006). A simple rule for the evolution of cooperation on graphs and social networks. Nature.

[29] Peleteiro, A., Burguillo, J. C. & Chong, S. Y. Exploring indirect reciprocity in complex networks using coalitions and rewiring. *International Conference on Autonomous Agents & Multiagent Systems*, Paris, France, 669–676 (2014).

[30] Pinheiro FL, Hartmann D (2017). Intermediate levels of network heterogeneity provide the best evolutionary outcomes. Sci. Rep..

[31] Pinheiro FL, Pacheco JM, Santos FC (2012). From local to global dilemmas in social networks. PLoS ONE.

[32] Pinheiro FL, Santos FC, Pacheco JM (2016). Linking individual and collective behavior in adaptive social networks. Phys. Rev. Lett..

[33] Airiau S, Sen S, Villatoro D (2014). Emergence of conventions through social learning. Auton. Agents Multi Agent Syst..

[34] Zhao K, Yen J, Ngamassi LM (2012). Simulating inter-organizational collaboration network: A multi-relational and event-based approach. SIMULATION.

[35] Agranoff R (2008). Enhancing performance through public sector networks: Mobilizing human capital in communities of practice. Public Perform. Manag. Rev..

[36] Dyer JH, Nobeoka K (2000). Creating and managing a high-performance knowledge-sharing network: The Toyota case. Strateg. Manag. J..

[37] Knight L (2002). Network learning: Exploring learning by interorganizational networks. Hum. Relat..

[38] Reiter, J. G., Hilbe, C. R., Rand, D. G., *et al*. Crosstalk in concurrent repeated games impedes direct reciprocity and requires stronger levels of forgiveness. *Nat. Commun.*, **9(1),** article No. 555 (2018). 10.1038/s41467-017-02721-8 PMC5803203 29416030

[39] Hongyu Z, Jianqiang W, Hua Ma (2013). Grouping approach of learning team based on social network analysis and multidimensional feature clustering. Appl. Res. Comput..

[40] Chen CM, Chang CC (2012). Mining learning social networks for cooperative learning with appropriate learning partners in a problem-based learning environment. Interact. Learn. Environ..

[41] Jianye Yu, Yuanzhuo W, Xiaolong J (2018). Evolutionary analysis on information sharing behavior in social networks based on social evolutionary game. Acta Electron. Sin..

[42] Jiaqin S, Ruguo F, Ming L (2018). The evolution of cooperation in spatial prisoner's dilemma game with dynamic relationship-based preferential learning. Physica A.

[43] Holbrook RL, Kulik CT (2001). Customer perceptions of justice in service transactions: The effects of strong and weak ties. J. Organ. Behav..

[44] Louch H (2000). Personal network integration: Transitivity and homophily in strong-tie relations. Soc. Netw..

[45] Erdos P, Renyi A (1959). On random graphs. Publicationes Mathematicae.

[46] North DC (1994). Economic performance through time. Am. Econ. Rev..

[47] Barro RJ (2000). Inequality and growth in a panel of countries. J. Econ. Growth.

[48] Taylor PD, Jonker LB (1978). Evolutionarily stable strategy and game dynamics. Math. Biosci..

[49] Smith JM (1982). Evolution and the Theory of Games.

[50] Foster D, Young P (1990). Stochastic evolutionary game dynamics. Theor. Popul. Biol..

[51] Ritzberger K, Weibull JW (1995). Evolutionary selection in normal-form games. Econom. Econom. Soc..

[52] van Damme, E. E. C. & Weibull, J. Evolution in games with endogenous mistake probabilities. Other publications TiSEM 1c779ce9-9daa-4893-9ddb-e, Tilburg University, School of Economics and Management. (2002)

[53] Kaniovski YM, Young HP (1995). Learning dynamics in games with stochastic perturbations. Games Econ. Behav..

[54] Lee JH, Iwasa Y, Dieckmann U, Sigmund K (2019). Social evolution leads to persistent corruption. Proc. Natl Acad. Sci. USA.

[55] Hilbe C, Chatterjee K, Nowak MA (2018). Partners and rivals in direct reciprocity. Nat. Hum. Behav..

[56] Schmid L, Chatterjee K, Hilbe C (2021). A unified framework of direct and indirect reciprocity. Nat. Hum. Behav..

[57] Hauser OP, Hilbe C, Chatterjee K (2019). Social dilemmas among unequals. Nature.

[58] Martinangeli AFM, Martinsson P (2020). We, the rich: Inequality, identity and cooperation. J. Econ. Behav. Organ..

[59] Wang X (2022). Dryad Dataset.

[60] Holme P, Kim BJ (2002). Growing scale-free networks with tunable clustering. Phys. Rev. E.

[61] Binmore K (2005). Natural Justice.

[62] Szolnoki MP (2014). Evolution of extortion in structured populations[J]. Phys. Rev. E.

[63] Hanaki N, Peterhansl A, Dodds PS (2007). Cooperation in evolving social networks. Manag. Sci..

[64] Han X, Cao S, Shen Z (2016). Emergence of communities and diversity in social networks. SSRN Electron. J..

[65] Stopczynski A, Sear V, Sapiezynski P (2014). Measuring large-scale social networks with high resolution. PLoS ONE.

[66] Baer M (2010). The strength-of-weak-ties perspective on creativity: A comprehensive examination and extension. J. Appl. Psychol..

[67] Iwata M, Akiyama E (2016). Heterogeneity of link weight and the evolution of cooperation. Phys. A Stat. Mech. Appl..

[68] Den Haan WJ (1997). Solving dynamic models with aggregate shocks and heterogeneous agents. Macroecon. Dyn..

[69] Guimerà R, Uzzi B, Spiro J (2005). Team assembly mechanisms determine collaboration network structure and team performance. Sci. Am. Assoc. Adv. Sci..

[70] Axelrod R, Hamilton W (1981). The evolution of cooperation. Science.

[71] Balafoutas L, Nikiforakis N, Rockenbach B (2014). Direct and indirect punishment among strangers in the field. Proc. Natl. Acad. Sci..

[72] Boyd R, Richerson PJ (1988). Culture and the Evolutionary Process.

[73] Brandt H, Hauert C, Sigmund K (2003). Punishment and reputation in spatial public goods games. Proc. R. Soc. Lond. Ser. B Biol. Sci..

[74] Panchanathan K, Boyd R (2004). indirect reciprocity can stabilize cooperation without the second-order free rider problem. Nature.

[75] Nowak MA, Sigmund K (1998). The dynamics of indirect reciprocity. J. Theor. Biol..

[76] Alexander RD (1987). The Biology of Moral Systems.

[77] Hamilton WD (1964). The genetical evolution of social behavior. Parts I, II. J. Theor. Biol..

[78] Granovetter M (2005). The impact of social structure on economic outcomes. J. Econ. Perspect..

[79] Aiello LM, Joglekar S, Quercia D (2022). Multidimensional tie strength and economic development. Sci. Rep..

[80] Chetty R, Jackson MO, Kuchler T (2022). Social capital II: Determinants of economic connectedness. Nature.

[81] Melamed D, Simpson B, Montgomery B (2022). Inequality and cooperation in social networks. Sci. Rep..

